# Refractory drug-induced systemic small-vessel vasculitis with two varied extracutaneous manifestations: a case report and review of the literature

**DOI:** 10.1186/s13256-023-04174-8

**Published:** 2023-10-27

**Authors:** Mark Jovanovic, Miso Sabovic

**Affiliations:** 1 Department of Internal Medicine, University Medical Centre Ljubljana, Ljubljana, Slovenia; 2 Department of Angiology, University Medical Centre Ljubljana, Ljubljana, Slovenia; 3 Faculty of Medicine, University of Ljubljana, Ljubljana, Slovenia

**Keywords:** Case report, Clopidogrel, Immunomodulation, Refractory vasculitis, Ticagrelor

## Abstract

**Background:**

Clopidogrel and ticagrelor are rarely reported to cause vasculitis via drug hypersensitivity reaction, largely mediated by T cells and immunoglobulin E (IgE). Despite therapeutic advances, the etiology of refractory vasculitides remains incompletely understood. Recently, (non)immunological mechanisms bypassing T cells and IgE have been proposed to explain resistance to standard immunosuppressants. Herein, we report a case of refractory drug-induced systemic small-vessel vasculitis with varied extracutaneous manifestations and incorporate multiple sources of data to provide detailed accounts of complex (non)immunological phenomena involved in this case. Study objectives are to provide an insight about rare presentations of commonly used drugs, upgrade the pathophysiological concepts of drug-induced vasculitis, raise need for further investigation to define causes and risk factors for refractory vasculitis, and discuss most of the current knowledge suggesting novel therapeutic approaches to treat this vasculitis. To our knowledge, this is the first case of the two flares of systemic small-vessel vasculitis in a single patient in response to clopidogrel and ticagrelor exposure, respectively. However, this report is limited by attribution/observer bias.

**Case presentation:**

We herein report a 24-year-old Caucasian male student with a medical history of mild seasonal allergic rhinoconjunctivitis, tension-type headaches, posttraumatic arterial stenosis, and previous exposure to ibuprofen, acetylsalicylic acid, and mRNA coronavirus disease 2019 (COVID-19) vaccine who suffered largely from acute urticaria and dyspnea after 20 days of acetylsalicylic acid and clopidogrel introduction. A skin punch biopsy confirmed leukocytoclastic vasculitis. Serologic antibody testing, complement analysis, microbiologic testing, and cancer biomarkers revealed no abnormalities. Regarding the patient’s medical history, both acetylsalicylic acid and clopidogrel were exchanged for ticagrelor. Furthermore, the addition of naproxen, cyclosporine, bilastine, prednisolone, and montelukast resulted in complete recovery. After 7 days, diarrhea and hematuria occurred. Urinalysis and computed tomography showed reversible proteinuria with gross hematuria and hypodense changes in kidney medulla, respectively, associated with discontinuation of ticagrelor and naproxen. In addition, the patient recovered completely without any immunosuppression up-titration.

**Conclusions:**

This case highlights the role of clopidogrel and ticagrelor as possible triggering agents for systemic small-vessel vasculitis and offers an insight into novel therapeutic strategies for refractory vasculitides. Further research is needed to build on the findings of a current report.

## Background

Small-vessel vasculitis (SVV) is a clinicopathologic term defined by inflammation of small vessel wall (arterioles, capillaries, venules) and histopathologic evidence mostly consistent with leukocytoclasia, a perivascular inflammatory infiltrate largely composed of neutrophils with disintegration of nuclei into fragments [1–3]. This inflammatory process is precipitated by perivascular immunoglobulin (Ig) deposition (Gell and Coombs classification type III hypersensitivity reaction) [3–5], followed by activation of the immune cascade, including chemokine release [interleukins (IL), tumor necrosis factor (TNF)], adhesion and extravasation of leukocytes [6]. When leukocytoclasia presents the underlying perivascular inflammation, the histopathologic term leukocytoclastic vasculitis (LCV) is also used.

SVV often presents with cutaneous manifestations such as petechiae, nodules, urticaria and palpable purpura, the key clinical feature of this vasculitis [7]. In skin-isolated form, it is called cutaneous SVV. According to revised International Chapel Hill Consensus Conference (CHCC) nomenclature of vasculitides in 2012 [8], cutaneous SVV has been classified among single organ vasculitides, due to the prevalent involvement of the skin. Nevertheless, more recently, a Dermatologic Addendum to CHCC 2012 [2] updated the former classification, recognizing that cutaneous SVV could not only present as a skin component of systemic vasculitis, but also a single-organ vasculitis that differs with regard to clinical, laboratory, and pathologic features from recognized systemic vasculitides. According to the updated classification of SVV summarized in Table 1 [2], the skin rash does not present the mandatory criterium in the single-organ SVV diagnostic algorithm [6]. Indeed, Abukhatwah et al. and Park et al. have reported a case of IgA vasculitis without typical skin rash concomitant with c-antineutrophil cytoplasmic antibodies (c-ANCA) positivity [9] and Henoch-Schöenlein purpura without typical skin lesions [10], respectively. Furthermore, recent data from a large web-based electronic clinical record, the Rheumatic Diseases Portuguese Register (Reuma.pt), which included 701 patients with different vasculitis subtypes, revealed that the estimated prevalence of non-cutaneous single-organ vasculitides, including SVV, approximates 2% of all vasculitis cases [11]. This finding emphasizes the role of histologic confirmation on biopsy (often leukocytoclasia), the gold standard for a diagnosis of SVV (LCV). However, due to its rarity, more rigorous cross-sectional studies are scarce and difficult to conduct.

**Table 1:** Names for vasculitides adopted by the 2012 International Chapel Hill Consensus Conference on the Nomenclature of Vasculitides [2]

Large vessel vasculitis
Takayasu arteritis
Giant cell arteritis
Medium vessel vasculitis
Polyarteritis nodosa
Kawasaki disease
Small vessel vasculitis
ANCA-associated vasculitis • *Microscopic polyangiitis* • *Granulomatosis with polyangiitis (Wegener’s)* • *Eosinophilic granulomatosis with polyangiitis (Churg Strauss)*
Immune complex small vessel vasculitis • *Antiglomerular basement membrane disease* • *Cryoglobulinemic vasculitis* • *IgA vasculitis (Henoch-Schönlein)* • *Hypocomplementemic urticarial vasculitis*
Variable vessel vasculitis
Behcet’s disease
Cogan’s syndrome
Single-organ vasculitis
Cutaneous leukocytoclastic angiitis
Cutaneous arteritis
Primary central nervous system vasculitis
Isolated aortitis
Others
Vasculitis associated with systemic disease
Lupus vasculitis
Rheumatoid vasculitis
Sarcoid vasculitis
Others
Vasculitis associated with probable etiology
Hepatitis C virus-associated cryoglobulinemic vasculitis
Hepatitis B virus-associated vasculitis
Syphilis-associated aortitis
Drug-associated immune complex vasculitis
Drug-associated ANCA-associated vasculitis
Cancer-associated vasculitis
Others

Despite a considerable uncertainty due to the variability of its definition, the global prevalence of cutaneous SVV (LCV) is estimated to range from 3 to 30 cases per million people [12–14], although data are still lacking for many parts of the world, including the Indian subcontinent, China, Africa, and South America. In addition, it appears the incidence of several SVV subtypes, particularly ANCA-associated vasculitis, has increased fivefold in the last 30 years and approximates 4–10 cases per million people [14]. The reasons for increased incidence of ANCA vasculitis are not clear, but could be related to the increasing ease of ANCA testing. However, the comparison of SVV prevalence/incidence between different countries is limited since separating the influence of genetic and environmental factors in such epidemiological studies is difficult, as patients with different genetic backgrounds have been studied in different geographical locations.

Although cutaneous SVV appears to affect both sexes of all ages equally, some studies noted a slight predilection for older males [6, 15, 16]. In addition to sex predilection, data from one US population (Olmsted Country, Minnesota)-based retrospective study investigating a total of 84 patients (sex ratio approximates 1) with newly diagnosed skin-biopsy proven LCV revealed cutaneous SVV spreads systemically in 30–45% of cases, affecting multiple organs, including the musculoskeletal system, respiratory system, urogenital tract, gastrointestinal tract, and eye [13]. The most common subtypes of SVV associated with systemic involvement include immunoglobulin A vasculitis (IgAV), antineutrophil cytoplasmic antibody (ANCA)-associated vasculitis, cryoglobulinemic vasculitis, and connective tissue diseases and malignancies [6]. Therefore, SVV should be considered a syndrome rather than a specifically defined entity.

The etiology of SVV remains incompletely understood. The nature of this rare but complex disease is idiopathic in most cases (primary SVV). However, infections, neoplasms, and various medications have been identified as possible triggers for SVV (secondary SVV) [16]. It is estimated that drug-induced SVV, which is largely mediated by T cells and/or IgE via drug hypersensitivity reaction (DHR), accounts for up to 15% of vasculitis cases [17, 18]. The most reported drugs include penicillin, cephalosporins, sulphonamides, loop and thiazide diuretics, and nonsteroidal anti-inflammatory drugs (NSAIDs) [18]. In addition, recently reported agents involved in the induction of SVV include tumor necrosis factor (TNF) inhibitors [19], rituximab [20], tocilizumab [21], and immune checkpoint inhibitors (ICI) [22]. These findings fuel the hypothesis that alternative (non)immunological mechanisms bypassing T cells, a common therapeutic target of ICI, might be the underlying cause of this vasculitis. Therefore, proposed drug-induced immunomodulation (DII), rather than DHR, might explain the standard immunosuppressive treatment (SIT) failure, including corticosteroids, cyclosporine, antihistamines, and NSAIDs, in prevention of refractory SVV, which accounts for up to 10% of SVV cases [23]. Of note, these data are limited by single-center small-sample studies and further research is necessary for validation of refractory SVV prevalence. Nevertheless, Kolkhir et al. have recently performed a meta-analysis (involving 261 eligible studies) on SIT response in patients with urticarial vasculitis (UV) and demonstrated approximately 20% resistance to corticosteroid therapy, the most commonly used immunosuppressants in treatment of UV [16]. The authors hypothesized the above mentioned theory of (non)immunological mechanisms bypassing T cells (common target of corticosteroids) could partially explain the corticosteroid resistance in these patients. Recently, a large randomized, multicenter double-blind interventional clinical trial (BIOVAS) [24] started investigating the effectiveness of infliximab, rituximab, tocilizumab, and placebos in the treatment of refractory non-ANCA-associated vasculitis, and the results of this trial (expected till the end of 2025) could lead to more reliable conclusions on novel therapeutic strategies of refractory vasculitides.

The theory of (non)immunological mechanisms bypassing T cells and/or IgE that are involved in pathogenesis of vasculitis provides an insight into understanding the pathophysiological concepts of drug-induced vasculitis better, beyond the scope of DHR, which could be explained by DII. Although DHR and DII are sometimes used interchangeably, these two terms should be differentiated due to various immune pathomechanisms and also variable response to SIT used in treatment of patients with SVV [25, 26].

**Fig. 1:**
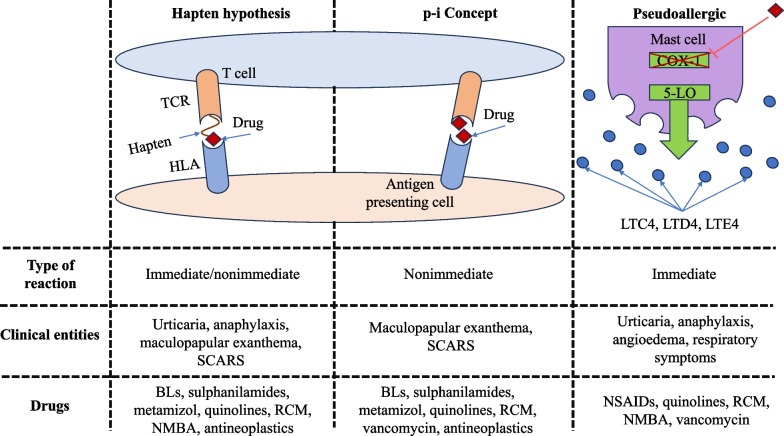
Mechanisms of drug interaction with immunological system in different types of DHRs. Adapted from Mayorga C et al. [27]. TCR, T-cell receptor; HLA, human leucocyte antigen; SCARs, severe cutaneous adverse reactions; BLs, beta lactams; RCM, radiocontrast media; NSAIDs, nonsteroidal anti-inflammatory drugs; NMBA, neuromuscular blocking agent; 5-LO, 5-lipoxygenase; LTC4, leukotriene C4; LTD4, leukotriene D4; LTE4, leukotriene E4; COX-1, cycloxigenase-1

DHR are based on distinct mechanisms and are clinically heterogeneous. They are broadly categorized into three forms based on the mechanism the drug interacts with immune cells; the allergic immune hypersensitivity via hapten formation (Gell and Coombs classification of immunological drug reactions), the p-i (pharmacological interaction with immune receptor) concept via drug interaction with immune cell receptor [human leucocyte antigen (HLA) or T-cell receptor (TCR)], and, finally, pseudo-allergy defined as drug interaction with receptors or enzymes of inflammatory cells without involvement of specific IgE or T cells (Fig. 1) [25, 27, 28]. Therefore, pseudo-allergic drug reactions are also considered as “non-immune hypersensitivity reactions” since the lack of clear evidence indicates that these reactions are driven by either humoral or T-cell-mediated immunological mechanisms [5]. Common drugs and agents that can cause pseudo-allergy are opioids, micelle-solubilized drugs (cyclosporine), NSAIDs (acetylsalicylic acid, naproxen), vancomycin, ciprofloxacin, and radiocontrast agents [18].

In comparison with DHR, which is associated with exaggerated immune reaction driven by T cells and/or IgE in response to exposure to a specific drug (Gell and Coombs classification of immunological drug reactions), DII is a term used to describe any drug actions on the immune system that result in either immune stimulation or suppression [26, 28]. Also, like pseudo-allergy reactions described above, DII involves drug-induced (non)immunological mechanisms without direct drug interaction on T cells that result in enhancement or attenuation of immune system and, therefore, could partially explain SIT failure in prevention of refractory vasculitis [16, 23].

Unlike beta lactams and NSAIDs, exposure to clopidogrel and/or ticagrelor is rarely associated with SVV [29–33]. Although the prescription of these two drugs has increased dramatically over the past decade for a variety of reasons, including the ageing of the population, the increase in cardiovascular morbidities, and the development of less invasive percutaneous revascularization procedures [34], the incidence of vasculitis cases associated with exposure to these drugs appears to be low [29, 31, 33]. It is estimated that any type of DHR occurs in 6% and 0.1–1% of patients receiving clopidogrel and ticagrelor, respectively [35, 36]. Although rarely reported, however, Mahgoub et al. and Seecheran et al. have reported severe systemic SVV with multiple organ failure after exposure to clopidogrel and ticagrelor [32, 33], and no clear factors have been recognized yet to identify patients at high risk for life-threatening SVV because of the rarity of this condition and the heterogeneous patient population [16]. Indeed, Sarkar et al. have recently demonstrated in a small-sample cross-sectional study the conventional biomarkers, such as the complement level, do not correlate well with disease severity; however, direct immunofluorescence might present a promising histopathologic method to identify patients with severe systemic SVV, particularly with gastrointestinal involvement [37]. In addition to undefined risk factors for severe systemic disease, there is a small risk of cross-reactivity between clopidogrel and ticagrelor, although these drugs belong to different chemical classes (clopidogrel to the thienopyridines and ticagrelor to the cyclopentyl-triazolo-pyrimidines) [32, 33, 38]. The cross-reactivity between clopidogrel and ticagrelor is rare, but clinically important since it requires regular follow-up of patients after introduction of and/or switch to each of these drugs.

We herein report a unique case of refractory drug-induced systemic SVV with two varied extracutaneous manifestations in response to exposure to multiple drugs, including clopidogrel and ticagrelor, in a single patient and discuss possible (non)immunological mechanisms involved in the pathogenesis of this vasculitis (DHR and DII), to explain SIT failure in prevention of the second flare. We also discuss the possible drug triggers for each flare of this vasculitis based on each clinical presentation and work-up performed. The objectives of this case study and observational research are to provide an insight about rare presentations of commonly used drugs, upgrade the pathophysiological concepts of drug-induced vasculitis beyond the scope of DHR, highlight the need for further investigation to define causes and risk factors for refractory vasculitis, and discuss most of the current knowledge suggesting novel therapeutic approaches to treat refractory vasculitides. To our knowledge, we believe this is the first reported case of the two consecutive systemic clinical presentations of SVV in a single patient in response to exposure to clopidogrel and ticagrelor, respectively. In addition, it alerts physicians on possible life-threatening adverse events in response to exposure to both clopidogrel and ticagrelor, despite low risk of cross-reactivity between these two drugs. However, this hypothesis is limited by attribution/observer bias and, also, lack of serologic/histopathologic evidence consistent with LCV in the second flare.

## Case presentation

### History

A 24-year-old Caucasian male student with a medical history of mild seasonal allergic rhinoconjunctivitis, tension-type headaches, and previous exposure to ibuprofen (regular treatment with ibuprofen 600 mg for 7 years due to chronic tension-type headaches), acetylsalicylic acid (ASA) (three-time exposure to ASA 100 mg before admission with no side effects reported), and the third dose of mRNA coronavirus disease 2019 (COVID-19) vaccine (received 64 days before admission with no side effects noted) was referred to our angiology department in March 2022 for posttraumatic stenosis of the right popliteal artery due to kickboxing injury. In our institution, percutaneous transluminal angioplasty was performed and dual antiplatelet therapy (DAPT) with ASA 100 mg and clopidogrel 75 mg was instituted after a thorough review of previous medical history, including allergy history. Also, the patient denied smoking and consuming illegal psychotropic drugs or anabolic steroids.

**Fig. 2:**
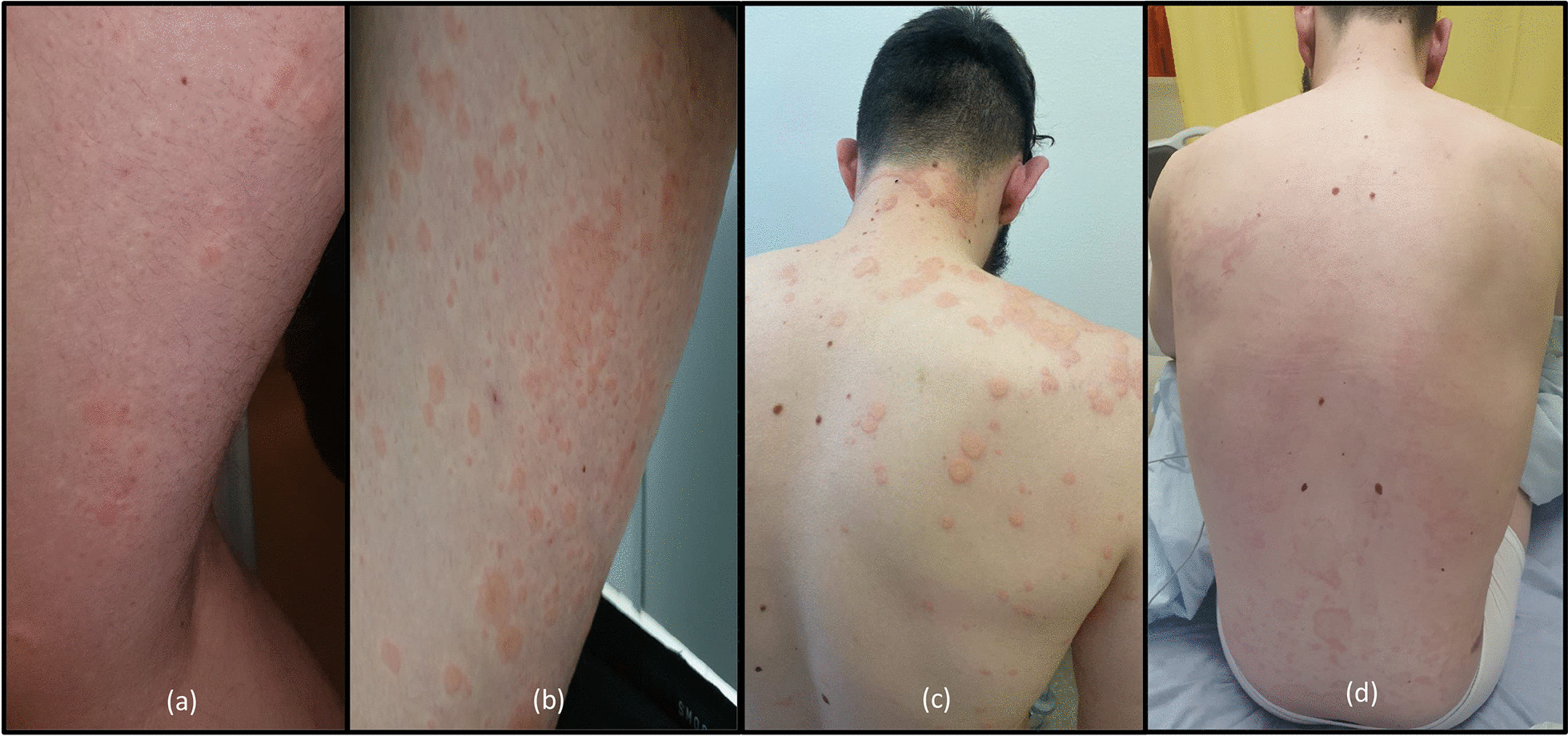
Patient presenting with a rash consisting of multiple, sharply demarcated erythematous plaques (urticaria) on the inner side of the right thigh after 20 days of ASA and clopidogrel introduction (**a**). The rash intensified within the next 24 hours (**b**) and spread to all limbs and the trunk within 72 hours (**c**). High-dose prednisolone treatment and discontinuation of both ASA and clopidogrel resulted in partial resolution of urticaria and dyspnea (**d**)

After 20 days of DAPT during his exchange program in a foreign country, a rash consisting of multiple sharply demarcated erythematous plaques (urticaria) appeared on the inner side of the right thigh and spread to the trunk and all limbs within the following 72 hours (Fig. 2a–d). The urticaria was accompanied by pruritus, and there were no bulges or erosions. Sun exposure also did not aggravate the rash. The rash was resistant to antihistamines and was complicated by the development of facial angioedema, symmetric arthralgia of metacarpophalangeal and interphalangeal joints, submandibular lymphadenopathy, and respiratory distress. During a physical examination, respiratory wheezes were auscultated as well. The chest radiograph on admission demonstrated few symmetric consolidations and reticulonodular opacities in both lungs, consistent with interstitial lung disease. He was admitted to the intensive care unit approximately 72 hours after the rash onset and was treated with supplemental oxygen, high-dose intravenous prednisolone (1 mg/kg per day) and bronchodilators including epinephrine, beta agonists, and anticholinergics. The initial response to treatment was good, leading to partial resolution of urticaria, arthralgia, and dyspnea, but he still needed a low flow rate of supplementary oxygen therapy. A skin punch biopsy was performed approximately 48–72 hours after the rash onset and the histopathologic pattern consistent with leukocytoclastic vasculitis (LCV) was confirmed. After evaluation of clinical presentation (urticaria) and histopathologic evidence of SVV (LCV), the diagnosis of urticarial vasculitis (UV) was set. Anticardiolipin immunoglobulin M (IgM), anti-beta2-glycoprotein I (anti-beta2GPI), antinuclear antibodies (ANA), antineutrophil cytoplasmic antibodies (ANCA), anti-proteinase-3 antibodies (anti-PR3), and anti-double-stranded DNA antibodies (anti-dsDNA) were negative. Normal C3 and C4 levels were also detected. In addition, no respiratory pathogens, hepatitis viruses, human immunodeficiency virus (HIV), *Treponema pallidum*, and Epstein–Barr virus (EBV) were detected. Also, no abnormalities were found in the blood count and inflammatory parameters were low. In addition, no malignant disease was confirmed. After clinicopathologic review of the UV with histopathologic evidence of SVV (LCV) and review of the patient’s medical history, including recent initiation of ASA and clopidogrel, both medications were discontinued and replaced with ticagrelor due to low potential of cross-reactivity between these drugs. In addition, cyclosporine, bilastine, prednisolone, and montelukast were administered, followed by complete resolution of patient’s symptoms, including urticaria, facial angioedema, lymphadenopathy, arthralgia, and dyspnea. Furthermore, naproxen was added to vasculitis treatment for pain management due to ibuprofen tolerance in the past. At discharge, the patient was prescribed with the following therapy: prednisolone 40 mg (reduced by 5 mg weekly to 20 mg per day), pantoprazole 40 mg, cyclosporine 100 mg + 100 mg + 150 mg, naproxen 550 mg/12 hours, bilastine (Opexa) 20 mg ×2 tablets every 12 hours, montelukast 10 mg, and ticagrelor 90 mg/12 hours.

After 7 days, the patient presented with abdominal distension, diarrhea, lymphadenopathy, and arthralgia, followed by gross hematuria over the next 5 days. He was referred to the emergency department, and an abdominal ultrasound revealed no bladder pathology and no evidence of hydronephrosis. However, abdominal computed tomography (CT) showed bilateral renal lesions suggestive of renal infarction, largely involving the kidney medulla (Fig. 2a). The patient was referred to our angiology department for further evaluation and treatment.

### Physical findings

On clinical examination, the patient was hemodynamically and respiratorily stable. The abdomen was diffusely tender and gross hematuria was present. We found no skin eruptions, but hands and ankles were slightly swollen and painful. Also, lymphatic nodes in both armpits were slightly tender.

### Laboratory data

Complete blood count revealed 11.80 × 109/L white blood cell count, with an increase in neutrophil granulocytes (11.09 × 109/L), but normal red blood cell and platelet counts. More extensive laboratory showed a serum creatinine of 0.67 mg/dL and a calculated glomerular filtration rate of > 90 mL/minute/1.73 m^2^ (Chronic Kidney Disease Epidemiology Collaboration; CKD-EPI). The erythrocyte sedimentation rate (ESR) was 11 mm, and C-reactive protein (CRP) was 18 mg/L. Alanine aminotransferase (ALAT) was slightly elevated (ALAT 29 U/L). Thyroid hormones were within normal range. Serum protein electrophoresis revealed decreased level of gamma-globulins (5.0 g/L, reference range 8.0–13.5 g/L), with normal level of alpha- and beta-globulins and albumins. The rheumatologic work-up, including rheumatoid factor (RF), lupus anticoagulant, anti-cardiolipin (a-CL) immunoglobulins, anti-beta2-glycoprotein I (anti-beta2GPI), antinuclear antibodies (ANA), antineutrophil cytoplasmic antibodies (ANCA), anti-proteinase-3 antibodies (anti-PR3), anti-myeloperoxidase antibodies (anti-MPO), and anti-double-stranded DNA antibodies (anti-dsDNA), was negative. Complement analysis showed normal C3 and C4 fractions (1.14 g/dL and 0.17 g/dL, respectively). Anti-C1q antibodies were < 20 U/mL. Hemostasis parameters, including activated partial thromboplastin time (APTT), prothrombin time (PT), thrombin time (TT), and fibrinogen, showed no abnormalities. Urinalysis revealed proteinuria 2+, hemoglobin 3+, severe erythrocyturia, and a white blood cell count of 15/µL. The percentage of dysmorphic erythrocytes was 2%. Urinalysis for 24 hours revealed proteinuria of 0.37 g. The level of cyclosporine was 69.6 ng/mL.

### Diagnosis and treatment

Because of the suspicion of renal infarction reported on the website abdominal CT, exclusion of possible thromboembolism was indicated. Therefore, standard heparin was administered empirically and ticagrelor was discontinued. In addition, naproxen was also discontinued due to renal impairment. After 7 days, the patient had fully recovered, and urinalysis showed no abnormalities. In addition, no SIT up-titration was performed. During the patient’s recovery, the dose of prednisolone was reduced according to predicted dosage regimen, and no relapse was reported.

To detect a possible thromboembolic focus, electrocardiography (ECG), echocardiography, and magnetic resonance angiography (MRA) of the thoracic aorta were performed. The ECG showed sinus rhythm with normal ventricular rate response, and echocardiography revealed normal-sized cardiac chambers without valvular pathology and normal systolic function without a clear cardiac shunt. In addition, MRA of the thoracic aorta and CT of the abdominal aorta showed no evidence of vasculitis, and no atherosclerotic plaques were detected. Also, magnetic resonance imaging (MRI) of the lung showed no clear pulmonary infiltrates noted on chest radiograph during the patient’s previous hospital stay (although CT thorax is more sensitive for detection of fine pulmonary infiltrates than MRI, we did not decide on the former imaging modality due to absence of respiratory symptoms and higher radiation dose). Furthermore, Doppler ultrasonography of renal vessels revealed normal kidney perfusion and slightly hypoechogenic changes in the kidney medulla consistent with acute kidney injury. These imaging findings suggested bilateral kidney lesions depicted on the abdominal CT were unlikely the consequence of thromboembolic event. Therefore, we discontinued anticoagulant therapy with standard heparin.

**Fig. 3:**
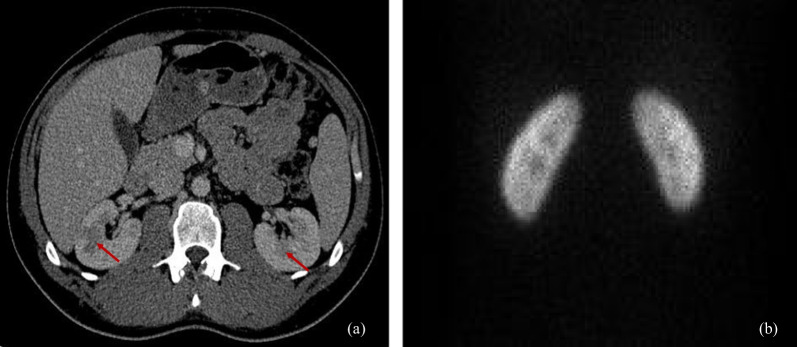
Normal-sized kidneys with multiple bilateral hypodense changes, largely involving the kidney medulla (left and right red arrows are pointing to hypodense changes suspected of renal infarction that measure 16 mm and 25 mm in diameter, respectively. (**a**) Static (cortical) renal scintigraphy showed complete regression of renal lesions visualized on abdominal CT (**b**)

Because of the reversible changes in urinalysis and the lack of signs of vasculitis on MRA and CT, we doubted the diagnostic efficacy of a renal biopsy. The latter is an invasive procedure associated with complications such as bleeding. Therefore, instead of a renal biopsy, a static (cortical) renal scintigraphy was performed, which showed complete regression of the renal lesions previously detected on the abdominal CT (Fig. 3b).

Due to transient abdominal distension and diarrhea, esophagogastroscopy, colonoscopy, and capsule endoscopy were performed and showed no abnormalities. In addition, ophthalmoscopy should be performed for detection of possible signs consistent with SVV (LCV); however, the patient denied any visual disturbance during his hospital stay and was referred to ophthalmic outpatient care.

According to the typical extracutaneous manifestation for UV (involvement of lungs, gastrointestinal tract, kidneys, joints, lymphatic nodes) presented in both flares, histopathologic evidence of SVV during the first flare, laboratory results, and imaging findings, the diagnosis of drug-induced SVV seemed most likely. The following hypothesis was supported by the fact that the patient recovered completely after discontinuation of ticagrelor and naproxen. However, this hypothesis is limited by lack of serologic/histopathologic evidence consistent with LCV during the second flare. In addition, language and/or cultural difference between the two hospitals the patients had been treated at presents potential bias among collected data from medical records.

The patient was discharged from our hospital on the 20th day of his hospital stay with the following therapy: prednisolone 20 mg, pantoprazole 40 mg, cyclosporine 100 mg + 100 mg + 150 mg bilastine (Opexa) 20 mg 2× tablets every 12 hours, and montelukast 10 mg.

**Fig. 4:**
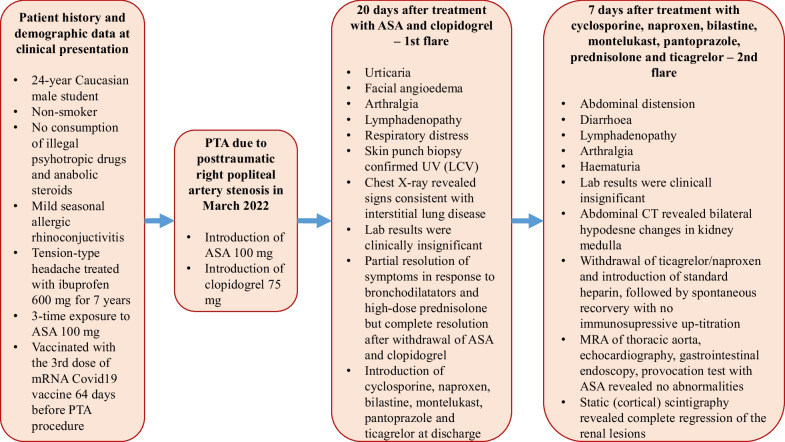
Diagram presents the case chronologically, including detailed patient history, initial symptoms and examination findings, test results, important diagnostic procedures, and treatment. ASA, acetylsalicylic acid; PTA, percutaneous transluminal angioplasty; UV, urticarial vasculitis; LCV, leukocytoclastic vasculitis

At the 9-month follow-up, the patient refused treatment with NSAIDs, as well as clopidogrel and ticagrelor, and successfully completed his SIT without complications, and no relapse was reported. At the 13-month follow-up, the patient performed provocation test with ASA and no adverse reactions were noted. Provocation test was performed to determine potential ASA hypersensitivity in case of the need for cardiovascular treatment in the future. However, the patient needs regular follow-up, since it is estimated the average duration of UV is 3–4 years with individual cases extending beyond 20 years [39]. Although normal complement levels might forecast benign clinical course of this vasculitis, the patient experienced significant morbidity with pulmonary involvement during the first flare. Indeed, there have been rare reports of fatal episodes of laryngeal edema and pulmonary hemorrhage due to exacerbation of UV (SVV) [40, 41]. However, since the complete resolution of dyspnea and absence of clear pulmonary infiltrates on chest MRI, further pulmonary evaluation during patient’s hospital stay was not performed (if persistent dyspnea was the case, chest radiography and pulmonary function testing would have been performed), and the patient was referred to pulmonary outpatient clinic. Of note, possible adverse reactions to SIT, such as acne, hypertension, hyperglycemia, weight gain, glaucoma, higher risk for infection, osteoporosis, neutropenia, and kidney dysfunction, should be regularly examined at out-patient visits [16]. Figure 4 presents the case chronologically, including detailed patient history, initial symptoms and examination findings, test results, important diagnostic procedures, and treatment.

## Discussion and conclusions

UV, as in this case, is a clinicopathologic entity consisting of two elements: clinical manifestations of urticaria and histopathologic pattern consistent with leukocytoclastic vasculitis [1]. Even though it has been rarely observed, the pattern of lymphocytic vasculitis has been recognized in patients with UV [16, 42]; however, there is still not enough evidence to prove that the lymphocyte pattern is truly etiologically or clinically relevant [16]. Indeed, Massa et al. have suggested old lesions of SVV, including UV, may no longer demonstrate leukocytoclasia and may contain mainly lymphocytes around blood vessels [42]. Also, this consideration stresses the importance of timing when taking a biopsy in a dynamic vasculitic process to avoid misdiagnosis. Of note, UV can mimic chronic spontaneous urticaria, and the clinical distinction between these two conditions is difficult but important due to different therapeutic management of the two skin diseases. Therefore, biopsy confirmation, although time dependent, presents the key diagnostic procedure in differentiation between these two skin conditions. To supplement histopathologic examination, Puhl et al. have recently proposed a novel histopathological scoring system to improve the histopathologic discrimination between these two skin conditions [43]. However, these data are based on a small-sample study and further research is needed to validate this scoring system. Distinguishing features between UV and chronic spontaneous urticaria and differences in therapeutic management are summarized in Table 2 [44].

**Table 2:** Distinguishing features of common urticaria and urticarial vasculitis [44]

Feature	Common urticaria	Urticarial vasculitis
Description	Mainly pruritic	Painful, tender, burning, and/or pruritic
Persistence	Between 8 and 24 hours	Between 24 and 72 hours
Residual effects	None	Purpura or hyperpigmentation
Predilection	• Trunk • Extremities • Face	• Trunk • Extremities • Face • Lateral borders of hands and feet
Dermographism	Common	Rare
Fixed lesions	No	Yes
Common triggers	• Viral illness • Antibiotics • Immunizations	• Infections • Autoimmune processes • Neoplastic processes • Drugs
Treatment	• Discontinue any new or unnecessary medications • Combinations of H1 and H2 antihistamines may be helpful • Systemic steroids can be helpful in severe cases	• Discontinue any new or unnecessary medications • H1 and H2 antihistamines for treatment of pruritus • NSAIDs for treatment of arthralgias • Systemic steroids with/without dapsone for treatment of moderate disease • Additional immunosuppressants, including mycophenolate mofetil, methotrexate, and cyclosporine A, for treatment of severe systemic disease

Although the histopathologic examination of affected organs (kidneys, gastrointestinal tract) was not performed during the second flare (of note, the negative histopathologic examination should not rule out SVV when clinically suspected and biopsy is not performed on time), we believe the two flares with various extracutaneous manifestations are two different clinical presentations of the same autoimmune disease (refractory drug-induced SVV), as both correlate well with exposure to recently administered new drugs and presence of extracutaneous manifestation commonly seen in patients with SVV, particularly in UV (cutaneous and lung involvement in the first flare, gastrointestinal and kidney involvement in the second flare, and lymphadenopathy/arthralgia in both flares). In addition, the absence of skin eruption (the typical clinical sign of UV) during the second flare could be masked by mast cell stabilizers (antihistamines, montelukast). In addition, cases of SVV without typical skin rash have been reported [9, 10]. We discuss the possible drug triggers for this vasculitis and attempt to interpret clinical findings, including SIT failure in prevention of the second flare, in relation to existing literature. Also, we emphasize novelties and highlight recommendations for future research and clinical practice. Of note, our interpretation is limited by attribution/observer bias and, also, lack of serologic/histopathologic evidence consistent with SVV (UV) in the second flare.

### Laboratory and imaging findings supporting the possible underlying SVV during the second flare

At the onset of the second flare, the patient presented with mild leucocytosis, high neutrophil lymphocyte ratio (NLR) (NLR 23.6, neutrophils 11.09, lymphocytes 0.47 x 10^9/L) and relatively low inflammatory parameters [CRP 18 mg/dL, procalcitonin (PCT) < 0.6 μg/L]. Following spontaneous recovery, NLR decreased with rise in peripheral blood lymphocytes (4–34.2%) (Table 3). In addition, serum protein electrophoresis revealed decreased level of gamma-globulins (5.0 g/L, reference range 8.0–13.5 g/L) with normal alpha- and beta-globulins and albumins. As suggested by Li et al. [45] and Fu et al. [46], these findings might reflect a dynamic inflammatory process inside a vessel wall, including deposition of gamma-globulins/immunoglobulins (low level of serum gamma-globulins) in the affected organs (kidneys, gastrointestinal tract, joints) and immunoglobulin-induced neutrophil activation (neutrophilia indicating possible leukocytoclasia in small vessel walls), followed by activation and migration of monocytes and lymphocytes (lymphocytosis indicating possible lymphocytic transformation of perivascular infiltrate) (Table 3) [45]. Therefore, NLR presents a respective inflammatory parameter as a potential supplementary diagnostic criterion in diagnosing SVV when there is a lack of serologic/histopathologic evidence and could serve as a useful biomarker to predict gastrointestinal and renal complications in a subgroup of patients with SVV (IgA vasculitis)—a finding based on data from meta-analysis [46]. However, further research is warranted to validate this parameter in patients with other types of SVV.

**Table 3:** Dynamics of complete blood cell count and urinalysis at admission and the 7th day of patient’s hospital stay

Parameter	At admission	7th day
Leukocytes (10^9^/L)	11.8	7.8
Neutrophils (%)	94.0	55.1
Monocytes (%)	1.0	6.3
Lymphocytes (%)	4.0	34.2
Eosinophils (%)	0.0	0.5
Basophils (%)	0.0	0.5
Erythrocytes (10^12^/L)	4.1	4.3
Hemoglobin (g/L)	122	128
Platelets (10^9^/L)	261	216
Urine proteins ( +)	2	0
Urine hemoglobin ( +)	3	0
Urine nitrites ( +)	0	0
Leukocytes in urine sediment (10^6^/L)	6–15	0
Erythrocytes in urine sediment (10^6^/L)	Numerous, isomorphic	2

Of note, our interpretation also has several limitations, including both attribution and publication bias. Furthermore, SIT during the second flare confounds the interpretation of inflammatory parameters [47]; however, absence of SIT up-titration during the second flare does not completely explain spontaneous recovery, including decrease in leukocytes/neutrophils and resolution of hematuria (Table 3 depicts dynamics in urinalysis). Interestingly, transient isomorphic or non-glomerular erythrocyturia was consistent with reversible CT-depicted hypodense lesions involving largely the kidney medulla. What is more, MRA of the thoracic aorta at the onset of the second flare did not reveal any signs of large vessel vasculitis, which further makes the diagnosis of SVV during the second flare more likely [48]. Although non-nephrotic proteinuria confounds the interpretation of peripheral edema, serum albumin was inside normal range, which supports the hypothesis that arthralgia and peripheral edema were likely the presentation of SVV (UV). In serology work-up for vasculitides, anti-Sjögren’s antibodies (SSA/SSB) and cryoglobulins were not measured, but the patient did not show any clinical findings consistent with Sjögren syndrome, and hepatitis C virus (HCV) serology was negative [49].

### NSAIDs as possible triggers for SVV

NSAIDs, such as ASA and naproxen, could present the possible triggers for both flares driven by DHR (type III), as the patient’s symptom onset and recovery were related to the introduction and discontinuation of these NSAIDs (10% of drug-induced vasculitides) [18]. Regarding previous exposure to ASA with no side effects reported, sensitization to ASA and cross-reactivity with naproxen could explain possible NSAID-induced SVV. This hypothesis is supported by further progress in understanding hypersensitive reactions to non-steroidal anti-inflammatory drugs, including increased risk of NSAID-exacerbated respiratory disease (NERD) and NSAID-induced urticaria/angioedema (NECD) among patients with underlying rhinoconjunctivitis [50]. However, the onset of these pseudo-allergic reactions is expected to start immediately after exposure to NSAIDs [27], which is not the case in this clinical course and therefore makes NERD and/or NECD less likely. Also, ASA provocation test was negative at 13-month follow-up when the patient completed SIT, but its diagnostic value is limited to delayed reactions occurring more than 24 hours after drug intake [51]. Also, despite the reported high cross-reactive hypersensitivity between ASA, naproxen, and ibuprofen (non-allergic COX-1-inhibition contributes to the majority of cases) [52, 53], the second flare cannot be completely explained by the exposure to naproxen alone since the patient never reported any symptoms in the last 7 years after taking high doses of ibuprofen. Nevertheless, the patient received the third dose of mRNA COVID-19 vaccine (no adverse effects reported) 64 days before the onset of the first flare, which might present a risk factor for evolution of autoimmune diseases such as UV (SVV) in response to exposure to drugs such as NSAIDs. Indeed, recent findings from meta-analysis highlight a link between a severe acute respiratory syndrome coronavirus 2 (SARS-CoV-2) vaccination and new onset or worsening of inflammatory and autoimmune skin diseases [54]. However, further research is warranted for its validation.

### Clopidogrel and ticagrelor as possible triggers for SVV

Although clopidogrel and ticagrelor belong to different chemical classes (clopidogrel to the thienopyridines, and ticagrelor to the cyclopentyl-triazolo-pyrimidines), small risk of cross-reactive hypersensitivity between these drugs exists [32, 33, 35, 36, 38]. Indeed, Depta et al. speculate that cross-reactive hypersensitivity between these two drugs may be B cell- rather than T cell-mediated, as the former is more problematic for drugs, with similar localized stereochemical similarities but significant structural core differences [55]. This speculation on immunological mechanism bypassing T cells partially explains SIT failure in prevention of the second flare; however, this is not the case for the first flare since the patient had completely recovered soon after introduction of SIT (especially cyclosporine) with its largely immunosuppressive effects on T cells [56, 57]. Therefore, immunological mechanisms largely mediated by T cells (DHR), rather than cross-reactive hypersensitivity between clopidogrel and ticagrelor mediated by B cells, appears more likely to be involved in the first flare of this vasculitis. Of note, this hypothesis suggests different immunological mechanisms are underlying each flare. However, according to our hypothesis that these two flares present the same autoimmune disease (SVV or UV) due to the aforementioned reasons, both episodes are likely to share common pathophysiological (non-immunological) mechanisms that are involved in regulation of immune system and thus also explain different clinical presentations of each episode.

Consistent with existing literature, the current data addressed the role of purinergic receptors in lung microvascular endothelial cell barrier (LMECB) integrity [58, 59]. Indeed, Zemskov et al. and Kolosova et al. have demonstrated that adenosine triphosphate (ATP) can significantly enhance the human LMECB via P2Y4 and P2Y12 receptors, the main target of both clopidogrel and ticagrelor [59, 60]. Therefore, it fuels the hypothesis introduction that clopidogrel (antagonist of P2Y12 receptor) might lead to LMECB integrity loss, followed by activation of inflammatory process largely mediated by T cells in response to exposure to antigens of LMECB. An et al. have also reported this possible association between clopidogrel and LMECB integrity loss [61]; however, it lacks any strong scientific evidence. In addition, Terentes-Printzios et al. have demonstrated in a recently conducted small-sample prospective study of the BNT162b2 mRNA vaccine against COVID-19 that the vaccine induces a moderate and transient short-term dysfunction of the endothelium that is almost entirely reversed in 48 hours [62]. Although this time frame does not explain the onset of the first flare in this case, results of this study support the findings from meta-analysis [54] on the mRNA COVID-19 vaccine as a predisposing risk factor for evolution of autoimmune diseases via sensitization of endothelial cell antigens [54, 62]. However, further studies are needed to support this hypothesis.

In addition to LMECB integrity, purinergic receptors are also involved in kidney and gastrointestinal physiology [63, 64]. Indeed, Burnstock et al. [65] and Zhang et al. [66] have demonstrated that P2Y12 receptors localize in the intrinsic primary afferent neurons (type II AH) of gastrointestinal submucous and myenteric plexus, as well as in the basolateral membrane of kidney collecting duct cells, respectively. This theory of purinergic receptor involvement in gastrointestinal and kidney physiology explains the patient’s clinical presentation during the second flare after exposure to ticagrelor, a reversible P2Y12 receptor inhibitor. Furthermore, Rashid et al. have recently reported a case of severe diarrhea after exposure to ticagrelor, suggesting involvement of purinergic receptors [67]; however, this association lacks strong scientific evidence. In addition to gastrointestinal system, Vallon et al. and Zhang et al. have demonstrated the blockade of the P2Y12 receptor has a dual affect in relation to arginine vasopressin (AVP) and renal water balance: it increases the production of AVP in the hypothalamus and enhances the action of AVP in the collecting duct, leading to increased expression of aquaporins (AQP) on the surface of collecting duct epithelial cells and thus leads to increased water retention [64, 66]. Recently, the concept of AQP, defined as simple water channels, has been extended in the contemporary literature to several pathophysiological processes, including several autoimmune diseases (e.g., rheumatoid arthritis, neuromyelitis optica spectrum disorders, primary Sjögren’s syndrome), and the underlying autoantibodies to AQP may confer pathogenicity through various mechanisms, including leukocytoclasia [68]. Indeed, Liao et al. and Su et al. have demonstrated AQP-3 is most prominently expressed in the colon and the basolateral membrane of kidney collecting duct cells, respectively [69, 70]. Furthermore, the later onset of hematuria than the onset of gastrointestinal symptoms in the second flare also suggests the possible involvement of AQP, since the necessary time needed for *AQP* gene transcription and translation is induced by purinergic receptors [66]. Also, Ohman et al. have demonstrated the ticagrelor induction of ATP release from human red blood cells, followed by B-cell proliferation via activation of purinergic receptors in an experimental *in vitro* study [71]. Regardless of the lack of strong scientific evidence to validate this observation, B-cell mediated immune response largely explains SIT failure in prevention of the second flare. However, Kolkhir et al. have demonstrated in recent systematic review the ineffectiveness of antihistamines (especially H2) and montelukast in the treatment of most patients with UV, but approximately 25–75% effectiveness has been displayed with the use of corticosteroids, NSAIDs, and cyclosporine in these patients [16]. Additionally, in this systematic review, cyclophosphamide (a cell cycle phase non-specific alkylating agent suppressing both T and B cells) [16, 72], anakinra (IL-1 receptor antagonist suppressing both T and B cells) [73], and rituximab (anti-CD20 Ab mainly inhibiting B-cell proliferation) [74] have appeared to demonstrate improved efficacy in the treatment of patients with UV resistant to corticosteroids [16], which suggests involvement of (non)immunological mechanisms bypassing T cells in patients with refractory UV. Large randomized double-blind placebo-controlled studies are needed for validation of this hypothesis. The main observations supporting the role of clopidogrel and ticagrelor in the pathogenesis of the first and the second flare are summarized in Table 4 [58, 59, 63–66, 75–77]. However, interpretation of these findings lacks strong scientific evidence and is limited by attribution bias.

**Table 4:** The summary of the main observations supporting the role of clopidogrel and ticagrelor in the pathogenesis of the first and the second flare [58, 59, 63–66, 75–77]

Observation	1st flare	2nd flare
Suspected trigger	Clopidogrel	Ticagrelor
The main clinical presentation	Urticaria Respiratory distress	Bowel dysfunction Hematuria
Signs of common autoimmune disease	Lymphadenopathy Arthralgia	Lymphadenopathy Arthralgia
Possible explanation of the main clinical presentation by drug pharmacodynamics/pharmacokinetics	Irreversible P2Y12 receptor binding might extend the severity of LMECB integrity loss (resulting in respiratory symptoms) Irreversible P2Y12 receptor binding might increase receptor desensitization and thus decrease expression of AQP (absence of gastrointestinal and renal symptoms) Higher glomerular filtration rate might decrease the drug passing through peritubular capillary network (absence of collecting duct cell injury)	Reversible P2Y12 receptor binding might not affect LMECB to the degree where it loses its integrity (absence of respiratory symptoms) Reversible P2Y12 receptor binding might decrease receptor desensitization and thus increase expression of AQP (leading to gastrointestinal and renal symptoms) Lower glomerular filtration rate might increase the drug passing through peritubular capillary network (presence of collecting duct cell injury)
The possible leading underlying (non)immunological mechanism	DHR with involvement of IgE (urticaria) and T cells	DII bypassing IgE and T cells
Possible pleiotropic drug effects via P2Y12 receptors	LMECB integrity loss	Increased transcription and translation of *AQP* genes Induction of ATP release from human red blood cells and proliferation of B cells
Correlation between clinical and imaging/histopathologic findings	Urticaria—more histopathologic evidence consistent with UV Dyspnea—more reticulonodular opacities in both lungs	Isomorphic (non-glomerular) erythrocyturia—more hypodense changes largely involving kidney medulla Absence of large vessel vasculitis signs during hematuria
Failure of standard immunosuppressive treatment	No	Yes

### Standard immunosuppressive treatment as possible trigger for SVV

SIT, including prednisolone, cyclosporine, montelukast, and bilastine, is the mainstay treatment of SVV (UV) in this case; however, SIT also presents as a potential trigger for the second flare of SVV (UV), although it is not completely understood why the patient has fully recovered without SIT up-titration. Indeed, Matsui et al. reported a case of montelukast-induced eosinophilic granulomatosis with polyangiitis, a subtype of SVV [78].

Corticosteroids (prednisolone) promote an anti-inflammatory state on both monocytes and macrophages, which might indirectly decrease the overall number and the activity of T cells via enhanced circulatory emigration [79], inhibition of IL-2 (a principal T-cell growth factor) [80] and induction of apoptosis [81, 82]. However, Olnes et al. have demonstrated in a small-sample (20 patients included) prospective study that corticosteroids do not cause significant acute changes in the overall numbers of human circulating B cells [56]. This study presents important background for further research to define corticosteroid immunosuppressive effects on B cells.

Cyclosporine is a lipophilic cyclic peptide that binds with high affinity to cyclophilins, a family of cytoplasmic proteins present in most cells. This drug–cyclophilin complex binds to and inhibits calcineurin, which leads to reduction in transcriptional activation of cytokine genes for IL-2, TNF-alpha, CD40L, GM-CSF, and INF-gamma [57]. Ultimately, proliferation of T lymphocytes is reduced. Despite the suboptimal therapeutic level of cyclosporine (69.6 ng/mL), the patient recovered completely without SIT up-titration. However, Savio et al. have suggested in systematic review that purinergic receptors might be involved in metabolism of prolyl-4-hydroxylase and its cofactor ascorbic acid, which present as important targets of cyclosporine in reduction of B cell activity [83]. Therefore, we hypothesize that ticagrelor counteracts with cyclosporine in the reduction of B cells via redox reactions [83]. However, this speculation needs further research work for its validation.

Mast cell stabilizers, including montelukast and bilastine, might mask the appearance of typical skin rash (urticaria) during the second flare due to suppression of histamine release from mast cells, one of the major mediators of most forms of urticaria [74]. However, Di Salvo et al. reported a case of montelukast-induced adverse skin reaction, including urticaria [84]; however, spontaneous recovery of patient’s symptoms without montelukast withdrawal is not completely understood and makes montelukast a less likely trigger in this case.

### Summary

Our discussion highlights the role of clopidogrel and ticagrelor as possible triggering factor for systemic SVV. In addition, the proposed underlying (non)immunological mechanisms involved in the pathogenesis of this refractory vasculitis present purinergic receptors and AQP as potential new therapeutic targets in patients with vasculitis resistant to SIT. Further large randomized double-blind placebo-controlled studies are needed to build on the findings of a current report.

## Conclusions

To our knowledge, this is the first reported case of two consecutive flares of systemic SVV in a single patient in response to clopidogrel and ticagrelor exposure, respectively. Therefore, it alerts physicians on possible life-threatening adverse events in response to both clopidogrel and ticagrelor, despite the low risk of cross-reactivity between these two drugs. In addition, this report provides an insight about rare but potentially life-threatening presentations of commonly used drugs, including clopidogrel and ticagrelor, and highlights the role of clopidogrel and ticagrelor as possible triggering factors for systemic SVV. This study upgrades the pathophysiological concepts of drug-induced vasculitis beyond the scope of drug hypersensitivity, raises the need for further investigation to define causes and risk factors for refractory vasculitis, and discusses most of the current knowledge suggesting novel therapeutic targets, including purinergic receptors and AQP, for treatment of vasculitides resistant to SIT. We hope this report will encourage researchers to continue the work on this complex but clinically significant topic and finally develop a more specific treatment for patients with refractory vasculitides.

## Data Availability

Data sharing is not applicable to this article as no datasets were generated or analyzed during the current study.

## Abbreviations

5-LO: 5-lipoxygenase
a-CL: Anticardiolipin immunoglobulin
ALAT: Alanine aminotransferase
ANA: Antinuclear antibodies
ANCA: Antineutrophil cytoplasmic antibodies
Anti-CD20 Ab: Cluster of differentiation 20 antibody
APTT: Activated partial thromboplastin time
anti-dsDNA: Anti-double stranded DNA antibodies
anti-PR3: Anti-proteinase-3 antibodies
AQP: Aquaporins
ASA: Acetylsalicylic acid
AVP: Arginine vasopressin
Anti-beta2GPI: Anti-beta2-glycoprotein I
BLs: Beta lactams
β-NAD: β-Nicotinamide adenine dinucleotide
CD40L: Cluster of differentiation 40 ligand
COX-1: Cycloxigenase-1
CRP: C-reactive protein
CT: Computed tomography
DAPT: Dual antiplatelet therapy
DHR: Drug hypersensitivity reactions
DII: Drug-induced immunomodulation
ECG: Electrocardiography
ESR: Erythrocyte sedimentation rate
HLA: Human leucocyte antigen
ICI: Immune checkpoint inhibitors
IL: Interleukins
INF: Interferon
IgAV: Immunoglobulin A vasculitis
LCV: Leukocytoclastic vasculitis
LT: Leukotriene
LMECB: Lung microvascular endothelial cell barrier
MRA: Magnetic resonance angiography
MRI: Magnetic resonance imaging
MPO: Myeloperoxidase enzyme
NLR: Neutrophil lymphocyte ratio
NSAID: Nonsteroidal anti-inflammatory drugs
NMBA: Neuromuscular blocking agent
PT: Prothrombin time
PTA: Percutaneous transluminal angioplasty
RCM: Radiocontrast media
RF: Rheumatoid factor
SCARs: Severe cutaneous adverse reactions
SIT: Standard immunosuppressive treatment
TCR: T-cell receptor
TNF: Tumor necrosis factor
TT: Thrombin time
UV: Urticarial vasculitis
